# Emotion Regulation, Positive Affect, and Promotive Voice Behavior at Work

**DOI:** 10.3389/fpsyg.2020.01739

**Published:** 2020-07-17

**Authors:** Hector P. Madrid

**Affiliations:** School of Management, Pontificia Universidad Católica de Chile, Santiago, Chile

**Keywords:** voice behavior, emotion regulation, positive affect, diary study, multilevel analysis

## Abstract

Promotive voice is an essential behavior in today’s organizations to facilitate improvements and make constructive changes in the way that work is conducted. Expanding previous research on the individual drivers of voice behavior in organizations, and drawing on theory about emotion regulation, I propose that speaking out with ideas at work is a function of employee emotion regulation and positive affect. Accordingly, results of a weekly diary study, conducted with professionals from diverse organizations and industries, showed that employees using emotion regulation strategies to improve their feelings increase the experience of positive affect at work, while behaviors oriented to worsen their own feelings were negatively related to the same outcome. Positive affect, in turn, increases the likelihood of promotive voice behavior. These results contribute to the voice behavior literature by showing that emotion regulation is an individual factor that participates in the construction of positive affective experiences, which is in turn conducive to speaking out with ideas for improvements and changes at work. Furthermore, these findings inform organizational practitioners about the value of training emotion regulation strategies to improve organizational effectiveness.

## Introduction

In the contemporary organization, employee behaviors such as actively proposing ideas to improve working methodologies, taking advantage of new opportunities in the environment, or preventing problems before they escalate are essential for organizational effectiveness. These behavioral processes have been described under the concept of voice behavior (Greenberg and Edwards, 2009). Given the importance of employee voice, determining which factors promote voice behavior has been a matter of extensive investigation (Morrison, 2014). As such, at the contextual level, for example, participation climate and psychological safety, together with supportive supervision and transformational leadership, have been found to facilitate speaking out with ideas in the workplace (Detert and Burris, 2007; Liu et al., 2010; Frazier et al., 2017). Furthermore, individual-level variables, such as job satisfaction, organizational identification, self-efficacy, and emotions, also promote or inhibit voice behavior (Edwards et al., 2009; Morrison et al., 2011; Liang et al., 2012). As part of this latter stream of research, scholars have paid a particular attention to affect because it has substantial influences on information processing and motivation (Forgas and George, 2001), as voice behavior involves judgments about the risks of speaking out and also willingness to go against the *status quo* in the work environment (Morrison and Milliken, 2000). Accordingly, diverse studies have concentrated on the role of negative emotions, showing that, for example, worry can lead to increased voice as it makes individuals concerned about and focused on achieving performance expectations, while anger can also mobilize the suggestion of ideas when this emotion is elicited by dissatisfaction with the current state of affairs (Edwards et al., 2009; Harvey et al., 2009; Madrid et al., 2015). In turn, fear decreases the likelihood of voice because it makes employees afraid of the possible negative consequences of going against the usual way of doing things at work, leading therefore to avoidant behavioral tendencies (Kish-Gephart et al., 2009).

Surprisingly, though, theory and research have paid less attention to whether states of positive affect could also play a role in voice behavior (Warr et al., 2014; Kirrane et al., 2017). This is an important omission since the affective experience in organizations is not limited to displeasure, but it also conveys enthusiasm, joy, and inspiration, and this sort of affect is conductive to positive attitudes and desirable behaviors (Shockley et al., 2012). Furthermore, research on voice behavior has paid less attention to the role of individual factors participating in the construction of affective experiences associated with the suggestion of ideas. Emotion regulation is one such neglected variable that may play an important role in employee voice. Emotion regulation involves a series of individual strategies oriented to provoke, sustain, change, and manage own emotions that have substantive impact on the way that individuals think and behave (Gross, 2013). I propose that promotive voice behavior, namely, the active suggestion of ideas to produce changes and improvements in the work environment (Liang et al., 2012), is a function of employee positive affect and emotion regulation behavior.

Emotion regulation entails a set of behaviors oriented to select and modify affect-eliciting situations, deploy attention from or reappraise affective-laden events, and modulate feelings deriving from these events (Gross, 1998). These behavioral strategies are an integral part of psychological functioning and adaptation, such that emotion regulation is linked to the experience of well-being and task performance in diverse performance domains (Gross and Thompson, 2007), including emotions, attitudes, and performance behavior in the workplace (Diefendorff et al., 2008; Lawrence et al., 2011). In this regard, expanding on the previous theory about emotion regulation, the model of Emotion Regulation of Others and Self (EROS) (Niven et al., 2009) proposes that individuals are motivated to improve or worsen their emotions. In the former case, people behave to reduce their unpleasant feelings or increase pleasant ones, whereas in the latter, they attempt to intensify negative affective experiences. As such, affect-improving emotion regulation behaviors involve cognitive reappraisal of affective experiences and also attention deployment by the use of distraction when confronted with a difficult event. In turn, affect-worsening emotion regulation is a form of affective dysregulation, in which individuals use cognitive rumination to enhance the experience of negative affect or reduce positive feelings (Niven et al., 2011). EROS also proposes that all of these regulation strategies are under the conscious control of the individual (controlled regulation) or are acting automatically beyond the individual’s awareness (automatic regulation).

Based on EROS, I propose that in the workplace, employee-controlled intrinsic emotion regulation behavior is associated with their experience of positive affect states at work. In general, emotion regulation behavior plays a central role in human adaptation by satisfying the process of approaching pleasure and avoiding pain (cf., Higgins, 1997). In this scenario, affect-improving emotion regulation, through the use of cognitive reappraisal expressed in, for example, thinking about positive aspects of the situation or thinking about one’s own positive characteristics when managing affective events, should be linked to positive feelings. This effect is likely due to cognitive reappraisal orientated to transforming negative experiences into positive meanings by modifying the way that situations are constructed and changing the subjective denotation of events (Brans et al., 2013; Katana et al., 2019). Furthermore, the distraction component of affect-improving emotion regulation may also lead to positive affect because it deploys attention from the negative contents of events when they happen or focuses attention on comfortable or joyful activities (Quoidbach et al., 2015). These strategies lead thereby to replacing negative thoughts and feelings with neutral or more positive psychological meanings (Brans et al., 2013; Brockman et al., 2017).

On the other hand, affect-worsening emotion regulation behavior, manifested in, for example, thinking of negative experiences or about one’s own shortcomings, should reduce positive affect of employees. These rumination processes focus attention on losses, failures, and adverse outcomes, involving the exacerbation of negative thoughts and a reduced sense of personal competence and self-efficacy (Nolenhoeksema et al., 1994; Lyubomirsky and Nolenhoeksema, 1995). Therefore, rumination often increases and sustains negative affect (e.g., depression and dysphoria) and lessens the experience of positive feelings, such as happiness and relaxation (Brans et al., 2013). As such, when individuals down regulate their feelings using affect-worsening regulation, they may experience less rewarding psychological states typically embedded in positive feelings, consuming also pleasure and energy.

In turn, positive affect states derived from emotion regulation should enhance the expression of promotive voice behavior. Positive feelings are rudiments of cognitive and behavioral processes unfolding in the workplace, such as job satisfaction, organizational citizenship behavior, proactivity, and innovation (Elfenbein, 2007; Bindl et al., 2012; Shockley et al., 2012; Madrid et al., 2014). These work-related outcomes are likely due to information processing and motivational processes associated with positive affect. Accordingly, positive feelings are related to flexible thinking and approach motivation (Watson, 2000; Fredrickson, 2001), which provides individuals with a broader perspective of their environment, a sense of safety in the given context, and willingness to pursue anticipated rewards in the environment (Higgins, 1997; Watson, 2000; Carver et al., 2008). This psychological configuration may be functional to the expression of promotive voice behavior because speaking out with ideas for change requires that employees have a comprehensive understanding of the work situation, a sense of safety to take the risk of defying the *status quo*, and readiness to manage resistance to change from coworkers (Morrison and Milliken, 2000; Madrid et al., 2015).

Thus, taking the above together, I hypothesize two mediational processes in which affect-improving emotion regulation increases voice behavior through enhanced states of positive affect, whereas affect-worsening regulation would decrease voice, due to the reduced experience of positive feelings.

Hypothesis 1: Affect-improving emotion regulation will be positively related to positive affect, which in turn will be positively related to voice behavior, such that positive affect mediates the positive relationship between affect-improving emotional regulation and voice.
Hypothesis 2: Affect-worsening emotion regulation will be negatively related to positive affect, which in turn will be positively related to voice behavior, such that positive affect mediates the negative relationship between affect-improving emotional regulation and voice.

Importantly, the relationships underlying these hypotheses should unfold over and above affective-laden personality traits of employees, namely, extraversion and neuroticism. These personality traits are predictors of contingent feelings, such as those comprising positive affect, because they describe tendencies to experience positive and negative feelings in the inner psychological realm (Eysenck, 1953). Measures of these traits highly overlap with measures of positive and negative trait affects (Watson and Clark, 1992). In turn, emotion regulation is about the management of feelings associated with these personality traits. Thus, I expect that affect-improving and -worsening emotion regulation behaviors exert incremental influences on positive affect, and thereby on voice behavior, relative to the increasing and lessening effects of extraversion and neuroticism, respectively.

## Methods

I conducted a weekly diary study to test the hypotheses, based on previous research showing that affect and change-oriented behavior are constructs that can fluctuate over weeks (Madrid et al., 2014). Thus, every Friday, across 4 weeks, participants provided ratings about the extent to which they experienced positive affect and enacted voice behavior in each respective week. One-week before starting the diary measures, participants responded to a survey with measures about their affect-improving and -worsening emotion regulation behaviors, together with extraversion and neuroticism. Participants were students of a part-time MBA program, who were also full-time employees in different organizations. To recruit these participants, they were sent an email with an invitation to be part of a study about emotion regulation and job performance. This email also described the procedure of the study and provided a URL link to register for the study. One hundred and fifty-two individuals completed the registration form to participate in the study, from which 125 participants answered the survey (82%). The gender of participants was 54% female, and their average age was 31.48 years (*SD* = 6.39). They were employees in organizations from manufacturing (6.5%), service (53.7%), consulting (3.3%), and other (36.6%) industries, in which their roles were administrative (20%), technical (1.7%), professional staff (54.2%), supervisor (11.7%), and manager (12.5%), while their average organizational tenure was 4.08 years (*SD* = 4.15).

Voice behavior was measured with three items from the scale developed by LePine and Van Dyne (1998) and adapted to measure this behavior on a weekly basis. Each participant was asked about the extent to which she/he, in the last week, has, for example, spoken up with ideas for new projects or changes in procedures (1: never – 5: many times, *α* = 0.87).

Positive affect was measured with three items from the scale developed by Warr et al. (2014), in which each participant indicated to what extent she/he, over the last week, has felt “enthusiastic,” “joyful,” and “inspired” (1: never – 5: almost always, *α* = 0.75).

Emotion regulation was measured with scales developed by Niven et al. (2011), in which participants provided information about the degree to which they enact, in general in their daily life, improving and worsening behaviors to regulate their emotions, with statements such as “I think of positive aspects of situations confronted,” “I do things I enjoy,” “I look for problems in my current situation,” and “I think about negative experiences” (1: not at all – 5: many times; *α* = 0.85 for affect-improving, *α* = 0.79 for affect-worsening).

Extraversion and neuroticism were measured with eight items from the scale developed by Benet-Martínez and John (1998), which asks each participant about the extent to which she/he is a person who, for example, “generates a lot of enthusiasm” and “worries a lot” (1: strongly disagree – 5: strongly agree; *α* = 0.81 for extraversion, *α* = 0.63 for neuroticism).

The data analysis strategy consisted of confirmatory factor analysis to determine the robustness of the measurement model underlying the study variables (Brown, 2006). Multilevel structural equation modeling (MSEM) was performed to test the hypotheses. In this, voice behavior and positive affect were defined as within-subjects variables and emotion regulation behavior, extraversion, and neuroticism as between-subjects variables. Data were analyzed using the multilevel framework described by Preacher et al. (2010), using a 2-1-1 mediation model in which emotion regulation behaviors (predictors) were defined at the level-2 and positive affect (mediator) and voice behavior (outcome) were defined at the level-1 (Preacher et al., 2010). To control for possible time serial dependence (auto-correlation) and monotonic time trend of voice behavior over waves of data, t-1 lagged factor of voice measures and the linear time index variable were included in the analyses.

## Results

**Figure 1:**
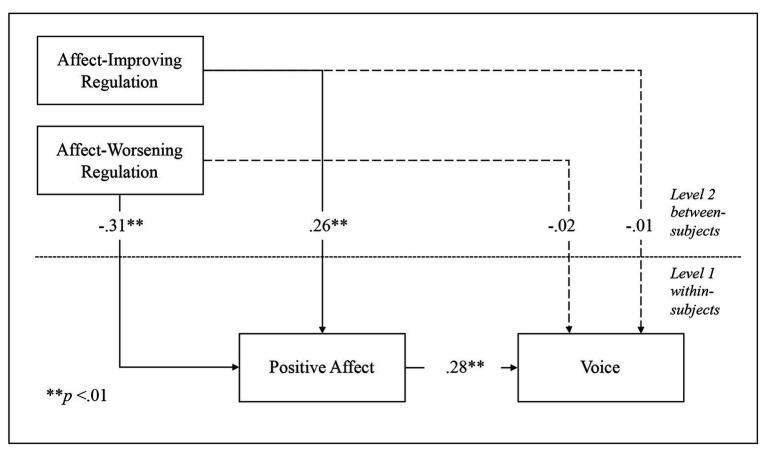
MSEM for voice, positive affect, and emotion regulation.

Results of confirmatory factor analysis showed acceptable goodness-of-fit, *χ*^2^(*df*) = 271.45(137), *p* < 0.01; RMSEA = 0.05, CFI = 0.91, supporting the robustness of the measurement model defined by voice, positive affect, affect-improving and -worsening emotion regulation behaviors, together with extraversion and neuroticism. Means, standard deviations, and correlations are presented in Table 1. MSEM showed that, over and above extraversion and neuroticism, affect-improving emotion regulation behavior was positively related to positive affect, *b* = 0.26, *SE* = 0.07, and *p* < 0.01, which in turn was positively related to voice behavior, *b* = 0.28, *SE* = 0.07, and *p* < 0.01. Furthermore, a positive indirect effect of affect-improving emotion regulation behavior on voice through positive affect was also observed, *b* = 0.07, *SE* = 0.03, and *p* < 0.05 (Table 2, Figure 1). Thus, Hypothesis 1 was supported. In turn, affect-worsening emotion regulation behavior was, over and above extraversion and neuroticism, negatively related to positive affect, *b* = −0.31, *SE* = 0.10, and *p* < 0.01, which, as described above, was positively related to voice behavior. In addition, a negative indirect effect was observed between affect-worsening emotion regulation and voice by means of positive affect, *b* = −0.09, *SE* = 0.04, and *p* < 0.05 (Table 2, Figure 1). Therefore, Hypothesis 2 was supported^1^.

**Table 1:** Means, standard deviations, correlations, and reliabilities.

Variable	M	SD	1	2	3	4	5	6
1. Extraversion	3.73	0.73	**(0.81)**					
2. Neuroticism	2.80	0.71	−0.25^**^	**(0.63)**				
3. Affect-improving	3.79	0.76	0.20^*^	−0.03	**(0.85)**			
4. Affect-worsening	1.27	0.48	−0.18^*^	0.35^**^	0.00	**(0.79)**		
5. Positive affect	3.53	0.76	0.46^**^	−0.25^**^	0.35^**^	−0.27^**^	**(0.75)**	0.46
6. Voice	3.83	0.79	0.33^**^	−0.24^**^	0.14	−0.21^*^	0.52^**^	**(0.87)**

*N*_between-subjects_ = 124–125, *N*_between-within_ = 399–404. Between-subjects correlations are lower the diagonal, within-subject correlations are upper the diagonal. Reliabilities are in bold and displayed in parentheses in the diagonal.

* *p* < 0.05,

** *p* < 0.01.

**Table 2:** Multilevel structural equation modeling (MSEM) for voice, positive affect, and emotion regulation.

Variable	Positive affect	Voice
*Intercept*	2.01 (0.42)^**^	1.42 (0.67)^*^
*Between-subjects effects*		
Extraversion	0.29 (0.07)^**^	0.01 (0.06)
Neuroticism	−0.05 (0.09)	−0.12 (0.06)
Affect-improving	0.26 (0.07)^**^	−0.01 (0.08)
Affect-worsening	−0.31 (0.10)^**^	−0.02 (0.06)
*Within-subjects effects*		
Time index		0.01 (0.03)
Lagged voice (t-1)		0.44 (0.16)^*^
Positive affect		0.28 (0.07)^**^
*Indirect effects*		
Affect-improving → positive affect → voice	0.07 (0.03)^*^	
Affect-worsening → positive affect → voice	−0.09 (0.04)^*^	
ICC	0.06	0.57
Deviance	1470.38	

*N*_within, between_ = 275/124. Unstandardized estimates, standard errors in parenthesis.

* *p* < 0.05,

** *p* < 0.01.

## Discussion

The results of this study showed that promotive voice behavior at work is a function of employee emotion regulation and positive affect. Employees who frequently use affect-improving emotion regulation strategies tend to experience enhanced positive feelings, such as enthusiasm, joy, and inspiration, increasing the likelihood of speaking out with ideas for improvements and changes. These effects are possible because cognitive reappraisal and distraction behaviors embedded in affect-improving emotion regulation facilitate the transformation of adverse experiences into a positive perspective, and they also direct the attentional focus to possible rewards available in the environment (Brans et al., 2013; Quoidbach et al., 2015; Brockman et al., 2017; Katana et al., 2019). In contrast, regular use of affect-worsening emotion regulation decreases voice behavior, due to its negative effect on positive feelings. This effect is given because this sort of emotion dysregulation involves cognitive rumination, a process focused on adverse experiences, losses, failure events, and negative thoughts (Nolenhoeksema et al., 1994; Lyubomirsky and Nolenhoeksema, 1995). In turn, the positive influence of positive affect on promotive voice behavior can be explained by the information processing and motivational correlates linked to feelings with positive valence (Watson, 2000; Fredrickson, 2001). This sort of affect broadens cognition, which provides a greater understanding of the task and work environment and also boosts the challenging and change-oriented behavioral tendencies often necessary for speaking out with alternative ideas. Importantly, these effects were over and above the influences of employee extraversion and neuroticism, highlighting that emotion regulation offers incremental validity in explaining why positive affect is experienced and can lead to valuable work behavior.

As such, this study contributes to the voice behavior literature by showing that speaking out in organizations is not limited to negative feelings (e.g., worry, anger, and fear), as previous studies have demonstrated (Morrison, 2014), because voice can also be a function of positive feelings. This finding also contributes to the literature on affect and work behavior in general, by adding to previous findings about the effects of positive feelings on, for example, citizenship behavior, proactivity, creativity, and innovation (Bindl et al., 2012; Shockley et al., 2012; Madrid et al., 2014). With regard to emotion regulation, previous research has explored the role played by emotional labor, a form of emotional regulation, in the expression of speaking up with ideas; however, the affective mechanisms mediating the relationship between these variables, to the best of my knowledge, have not been examined yet (Grant, 2013). Thus, another contribution of this study lies in supporting the participation of a set of self-regulation differences in the construction of positive work-related affective experiences linked to voice behavior. This contribution also encompasses the application of the emotion regulation literature, with a focus on the EROS model (Niven et al., 2009), to the work and organizational psychology domain.

Practical implications of the study’s results include that interventions oriented to the development of emotion regulation strategies with employees should be beneficial for voicing ideas at work and, therefore, potentially organizational effectiveness. Thus, the design and implementation of, for example, training initiatives to use affect-improving emotion regulation strategies and control enaction of affect-worsening emotion regulation behaviors could be beneficial for employee well-being and their performance. These strategies involve training in the use of cognitive reappraisal and distraction and the reduction of cognitive rumination. Furthermore, because positive affect is likely to facilitate speaking out with ideas, interventions to foster feelings such as enthusiasm, joy, and inspiration through, for example, job design, team building, leadership training, and management of work climate should encourage voice behavior.

Future research initiatives to expand knowledge developed in this study may explore whether affect-improving and -worsening emotion regulation behaviors interact with contextual characteristics or events in the prediction of affect and voice. For example, emotion regulation might be a boundary condition for the effects of stressor factors at the job, group, and organizational levels, manifested in, for instance, workloads, time pressures, and job complexity, together with dysfunctional group processes and perceptions of job unfairness and insecurity. Furthermore, emotion regulation strategies other than those studied here may be examined in relation to voice behavior, such as situation selection and response modulation (Gross, 2015). In contrast to cognitive reappraisal, distraction, and rumination, situation selection and response modulation act before and after affect-eliciting events, respectively, so these strategies may explain employees’ preparation to voicing their ideas, or how they emotionally react after they actually speak out.

This study is subject to a number of limitations. Causality assumed in the effects of emotion regulation on voice behavior through positive affect is only theoretically inferred, because the study relied on observational data collected using a survey design. Thus, for example, it might also be the case that voice behavior predicts positive affect because if proposed ideas are welcomed in the work environment, a sense of competence and personal accomplishment may be enhanced, which is often associated with positive feelings (Gagne and Vansteenkiste, 2013; Deci et al., 2017). Also, positive feelings might be the cause of affect-improving emotion regulation behavior, based on the human tendency to keep and sustain positive feelings over time (Higgins, 1997). Another limitation is associated with issues of common method variance in the statistical estimates modeled due to the use of self-reports in all the measures utilized, which might introduce bias in the results (Podsakoff et al., 2012). These issues are particularly sensitive to the relationship between positive affect and voice behavior, as this data, although based on repeated measures (Bolger and Laurenceau, 2013), were collected using the same survey at each measurement point. The use of employee extraversion and neuroticism as control variables helps to control these issues (Spector, 1994), but common method bias might remain in the model estimated. Therefore, future research should utilize experimental and longitudinal designs, based on multiple sources of information, to determine if the results observed here are robust and replicable.

To sum up, this study examined whether voice behavior at work is related to emotion regulation and positive affect, showing that improving and worsening own emotions, by means of positive affect, enhance or hinder speaking out ideas and suggestions at work. I trust that future research will expand knowledge developed here, which could also be informative for practical interventions to foster organizational effectiveness.

## Data Availability Statement

The datasets generated for this study are available on request to the corresponding author.

## Ethics Statement

The studies involving human participants were reviewed and approved by Ethics committee Pontificia Universidad Católica de Chile. The patients/participants provided their written informed consent to participate in this study.

## Author Contributions

The author confirms being the sole contributor of this work and has approved it for publication.

### Conflict of Interest

The author declares that the research was conducted in the absence of any commercial or financial relationships that could be construed as a potential conflict of interest.

## Footnotes

^1^Results excluding extraversion and neuroticism as control variables showed virtually the same.
